# Characteristics of very late-onset schizophrenia-like psychosis as prodromal dementia with Lewy bodies: a cross-sectional study

**DOI:** 10.1186/s13195-022-01080-x

**Published:** 2022-09-22

**Authors:** Hideki Kanemoto, Yuto Satake, Takashi Suehiro, Daiki Taomoto, Fuyuki Koizumi, Shunsuke Sato, Tamiki Wada, Keiko Matsunaga, Eku Shimosegawa, Mamoru Hashimoto, Kenji Yoshiyama, Manabu Ikeda

**Affiliations:** 1 Department of Psychiatry, Osaka University Graduate School of Medicine, D3 2-2 Yamadaoka, Suita, Osaka 565-0871 Japan; 2 Department of Molecular Imaging in Medicine, Osaka University Graduate School of Medicine, Osaka, Japan; 3 Department of Neuropsychiatry, Kindai University Faculty of Medicine, Sayama, Osaka Japan

**Keywords:** Very late-onset schizophrenia-like psychosis, Dementia with Lewy bodies, Imaging biomarkers, Psychomotor deterioration, Occipital hypoperfusion

## Abstract

**Background:**

This study aimed to identify cases of potential prodromal DLB in very late-onset schizophrenia-like psychosis (VLOSLP), using indicative biomarkers of dementia with Lewy bodies (DLB), and to evaluate the characteristics of psychosis as prodromal DLB.

**Methods:**

Data of patients with VLOSLP without dementia and Parkinsonism, who underwent testing for at least one indicative biomarker of DLB, were retrospectively collected from the database of the psychiatry clinic at the Osaka University Hospital. Patients were divided into two groups based on the positive (VLOSLP+LB) and negative (VLOSLP–LB) results of the indicative biomarkers of DLB. Age, gender, cognitive battery scores, prevalence of each type of delusions and hallucinations, cerebral volume, and cerebral perfusion were compared between the two groups.

**Results:**

Eleven VLOSLP+LB and 23 VLOSLP–LB participants were enrolled. There were no significant differences in age, proportion of females, and MMSE scores between the two groups. The standardized score of the digit symbol substitution test was significantly lower in the VLOSLP+LB than in VLOSLP–LB group (6.9 [3.1] vs. 10.0 [2.7], *p* = 0.005). The prevalence of visual hallucinations was significantly higher in the VLOSLP+LB group than in the VLOSLP-LB group (81.8% vs. 26.1%, *p* = 0.003). Auditory hallucinations were prevalent in both groups (43.5% in VLOSLP–LB, and 45.5% in VLOSLP+LB). Among patients with auditory hallucinations, auditory hallucinations without coexistent visual hallucinations tended to be more prevalent in VLOSLP–LB (7 out of 10) than in VLOSLP+LB patients (1 out of 5). Although cerebral volume was not different in any region, cerebral perfusion in the posterior region, including the occipital lobe, was significantly lower in the VLOSLP+LB group.

**Conclusions:**

Psychomotor slowing, visual hallucinations, and reduced perfusion in the occipital lobe may be suggestive of prodromal DLB in VLOSLP patients, even though the clinical manifestations were similar in many respects between VLOSLP+LB and VLOSLP–LB. Although auditory hallucinations were prevalent in both groups, most patients in VLOSLP+LB complained of auditory hallucinations along with visual hallucinations. Future studies with a larger number of patients without selection bias are desirable.

## Background

As the population ages, mental illness among older people is becoming a social problem globally [1]. Dementia is the most common example, and psychosis has become a major mental health problem among older people. Among older people, psychosis is frequently caused by dementia or delirium associated with physical illness. Primary psychosis, for which no organic cause has been identified, also occurs in older people. Such late-onset primary psychosis is known to have a different symptomatology from that of general, young-onset psychosis [2, 3]. In 2000, the international late-onset schizophrenia group defined late-onset schizophrenia as the onset of schizophrenia after the age of 40 years, and very late-onset schizophrenia-like psychosis (VLOSLP) was defined as an onset after the age of 60 years, independent of average age onset schizophrenia spectrum disorders [4].

Although VLOSLP was originally defined as primary psychosis, several studies reported that some patients with VLOSLP are associated with neurodegeneration, such as Lewy body disease (LBD), argyrophilic grain disease, and primary age-related tauopathy [5, 6]. In addition, some longitudinal studies reported that patients with VLOSLP frequently converted to dementia, as compared to the healthy controls [7–9]. In 2020, the research criteria for prodromal dementia with Lewy bodies (DLB) proposed a subtype of psychiatric-onset that presents with a condition that is diagnosed with psychiatric disorders, including psychosis, before presenting with dementia [10]. These previous studies indicate that some cases of VLOSLP could be associated with dementia-related neurodegenerative diseases, especially DLB. However, few studies have examined the presence of neurodegenerative pathology at the stage of diagnosis of VLOSLP before presenting with dementia, because neuropathological research is basically performed on postmortem brains.

Although the efficacy of antipsychotics for VLOSLP has been reported [11], hypersensitivity to antipsychotics is common in DLB. Therefore, understanding whether a patient with VLOSLP is in a prodromal state of DLB is important for implementing an effective treatment plan. With the recent development of biomarkers for neurodegenerative diseases, it is becoming possible to estimate the presence of Alzheimer’s disease (AD) and LBD. Reduced tracer binding on ^123^I-meta-iodobenzylguanidine (MIBG) myocardial scintigraphy and dopamine transporter imaging has been reported to be useful to predict evolution to DLB in subjects with mild cognitive impairment (MCI), major depression, and REM sleep behavior disorder (RBD) [12–17]. However, there are no reports on DLB biomarkers in VLOSLP, except for some case reports [18]. This study aimed to identify cases of potential prodromal DLB in VLOSLP using indicative biomarkers of DLB and to evaluate the characteristics of psychosis as prodromal DLB.

## Methods

### Study design

This study was a retrospective observational study without any intervention, and the information of all participants was anonymized prior to analysis as unlinked data to prevent the identification of personal information. The authors assert that all procedures contributing to this work comply with the ethical standards of the relevant national and institutional committees on human experimentation and with the Helsinki Declaration of 1975, as revised in 2008. The study was approved by the Research Ethical Committee of the Osaka University Hospital (Suita, Japan).

### Participants

We considered a retrospective cohort of non-demented patients with VLOSLP from the database of the neuropsychology clinic in the Department of Psychiatry at Osaka University Hospital, from January 2014 to March 2021, according to the criteria in the previous studies of VLOSLP [4, 11]. The inclusion criteria were as follows: (1) onset of delusions and/or hallucinations at an age of 60 years or more and (2) existence of delusions and/or hallucinations during the evaluation period. The existence of psychosis (delusions and/or hallucinations) was confirmed by the subitems of delusions and hallucinations in the Neuropsychiatric Inventory (NPI) if there was a reliable family informant or 30 points or more on the Brief Psychiatric Rating Scale (BPRS). The exclusion criteria were as follows: (1) cognitive impairment (Mini-Mental State Examination [MMSE] score < 24); (2) diagnosis of dementia (Clinical Dementia Rating [CDR] ≥ 1); (3) diagnosis of affective disorder; (4) comorbidity of Parkinsonism; (5) abnormal localized findings on magnetic resonance imaging (MRI), such as cerebrovascular disease or brain tumor; and (6) uncontrolled physical disorders that could cause psychosis.

### Assessment of clinical features

In our neuropsychological clinic, we assessed physical condition, demographic data, and medical history and performed standard neuropsychological examinations. In addition, the patients underwent routine laboratory tests and brain neuroimaging.

General cognition and memory were assessed using MMSE and logical memory in the Wechsler Memory Scale-Revised, respectively. In addition, attention, psychomotor speed, visuospatial cognition, and language were evaluated using the Digit Span, Digit Symbol Substitution Test (DSST), Block Design Test, and Information in Wechsler Adult Intelligence Scale-III (WAIS-III). These assessments were conducted by clinical psychologists and neuropsychiatrists specializing in geriatric psychiatry.

Neuropsychiatric symptoms and cognitive fluctuation were assessed using the NPI-plus [19], which is the original NPI-12 [20] with an additional subitem for cognitive fluctuation, if there was a reliable family informant. If there was no reliable family informant, psychosis was assessed using the BPRS. These assessments were also conducted by neuropsychiatrists specializing in geriatric psychiatry.

During data collection, cases lacking data on MMSE, CDR, and NPI or BPRS were excluded to select patients who met the inclusion criteria. Missing data on WAIS-III was allowed.

### Assessment of psychotic contents

We assessed the contents of psychosis according to sub-questions in the delusions and hallucinations section in the NPI. If there were no reliable family informants, the contents of psychosis were judged based on medical history and observation by the geriatric neuropsychiatrists. To evaluate the features of auditory hallucinations in DLB [21], we checked whether auditory hallucinations were accompanied with visual hallucinations (AH+VH) or not (AH–VH).

### Indicative biomarkers of DLB and group classification

We collected data from dopamine transporter single-photon emission computed tomography (DAT SPECT) using ^123^I-labeled N-ω-fluoropropyl-2β-carbomethoxy-3β-[4-iodophenyl] nortropane and MIBG myocardial scintigraphy to evaluate the indicative biomarkers of DLB. We judged the results according to the cutoff values of the specific binding ratio for DAT SPECT [22] and heart-to-mediastinum ratio for MIBG myocardial scintigraphy [23]. Patients who underwent testing for indicative biomarkers of DLB were divided into two groups according to whether the results were positive (VLOSLP+LB) or negative (VLOSLP–LB).

### Image acquisition and preprocessing of MRI data

We collected the following MRI data when available. MRI was performed using a l.5T system (Signa Excite HD 12.x, General Electric Medical Systems, Milwaukee). We acquired a three-dimensional volumetric T1-weighted gradient echo sequence to produce a gapless series of thin sagittal sections covering the whole brain. The operating parameters were as follows: field of view = 240 mm; matrix = 256 × 256; 124 × 1.40 mm contiguous sections; repetition time = 12.55 ms; echo time = 4.20 ms; and flip angle = 15°.

The individual MR images were segmented using “New segment” implemented in Statistical Parametric Mapping (SPM) version 12 (Welcome Department of Cognitive Neurology, London, UK) running under MATLAB R2018b (MathWorks Inc., Sherborn, MA) and transformed into a standard stereotaxic anatomical space with the brain template from the Montreal Neurological Institute (voxel size: 1.5 mm × 1.5 mm × 1.5 mm) using diffeomorphic anatomic registration through exponentiated lie algebra (DARTEL) flow created in the DARTEL process.

### Image acquisition and preprocessing of SPECT data

N-isopropyl-p-[^123^I] iodoamphetamine (^123^I-IMP) SPECT was conducted using an integrated SPECT/CT system (Symbia T-6; Siemens Healthcare, Erlangen, Germany) to evaluate cerebral blood flow. Studies were conducted in a resting state with eyes closed and ears unplugged. A 167 MBq dose of ^123^I-IMP was injected into the antecubital vein. The SPECT scan was initiated 15 min after the ^123^I-IMP injection, and data acquisition was performed for 30 min in dynamic mode with gamma cameras rotating over a 360° range in 4° angular steps (90 views). For image reconstruction, the scatter component in the projection data was estimated using the triple-energy window method. Scatter-subtracted tomographic data were applied for the filtered back-projection with Chang’s attenuation correction method with a 0.15 cm^−1^ attenuation coefficient.

### Statistical analyses

To evaluate what kind of patients with VLOSLP were examined for indicative biomarkers of DLB, the characteristics were compared between patients who had been tested and those who had not. The characteristics of VLOSLP+LB and VLOSLP-LB were also compared. For the aforementioned comparisons, we used the Mann–Whitney *U* test for continuous and ordinal variables and the chi-squared test for nominal variables. These analyses were performed using SPSS for Mac version 25.0 (IBM Corp., Armonk, NY, USA). The level of statistical significance was set at a two-tailed *p* < 0.05.

Differences in regional gray matter volume were examined using a two-sample *t*-test model in SPM between VLOSLP+LB and VLOSLP–LB. The specific effect of each parameter was tested using [1] or [− 1] *t*-contrast with additional zeros for the remaining nuisance covariates. Age, gender, and intracranial volume were used as nuisance covariates. The statistical threshold was set to an uncorrected *p* < 0.01, and the extent threshold was set at more than 300 voxels.

To compare cerebral blood flow over the entire brain between VLOSLP+LB and VLOSLP–LB, three-dimensional stereotactic surface projection (3D-SSP) images were firstly created using the Neurological Statistical Image Analysis Software (NEUROSTAT) [24]. Image analysis was performed using iSSP version 3.5 on FALCON version 6.1.0.0 (Nihon Medi-Physics Co., Ltd., Japan). In this process, the spatial distribution of abnormal cerebral blood flow was calculated for each SPECT data using the age-gender-matched normal control database of Osaka University Hospital. The spatial distribution of abnormal cerebral blood flow was compared between VLOSLP+LB and VLOSLP–LB by calculating two-sample *t*-statistic values (converted to *Z*) at each voxel using iSSP3.5_2tZ (Nihon Medi-Physics Co., Ltd., Japan). The statistical *Z*-threshold was set at 1.

## Results

### Characteristics

**Fig. 1:**
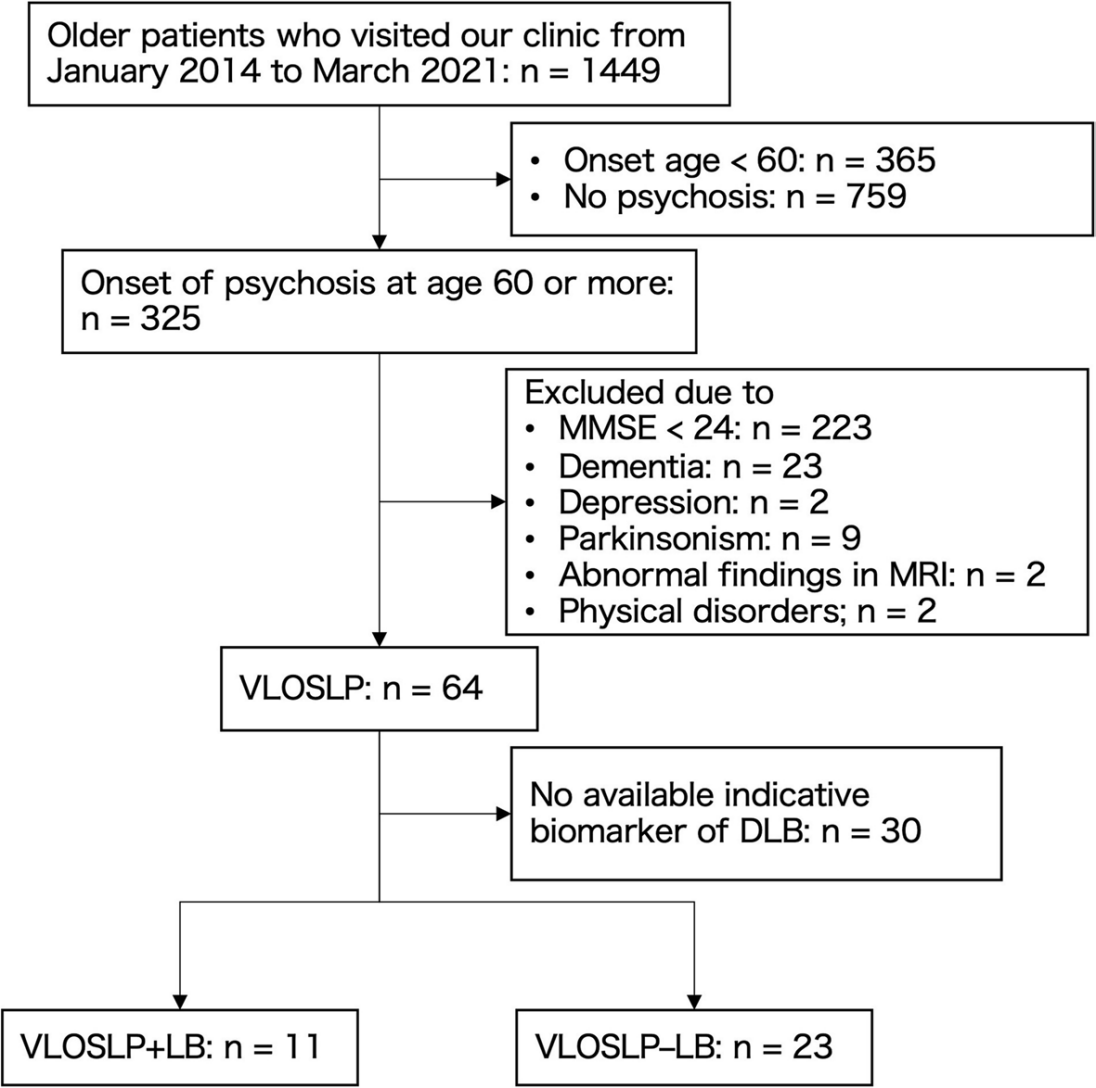
Participant selection. VLOSLP, very late-onset schizophrenia-like psychosis; DLB, dementia with Lewy bodies; VLOSLP+LB, VLOSLP with positive results in indicative biomarkers of DLB; VLOSLP–LB, VLOSLP with negative results in indicative biomarkers of DLB; CDR, Clinical Dementia Rating; MMSE, Mini-Mental State Examination

A total of 16 men and 48 women with VLOSLP were included (Fig. 1). The mean (SD) age and MMSE scores were 78.5 (6.4) and 26.4 (1.8), respectively (Table 1). Two patients had no reliable family caregiver; therefore, their psychotic symptoms were assessed using the BPRS (scores were 45 and 30). Forty-nine patients (76.6%) had delusions, 35 (54.7%) had hallucinations, and 20 (31.3%) had both. The most frequently observed delusions were persecution delusions (40.6%), followed by theft delusions (39.1%), and phantom-border delusions (31.3%). The most frequently observed hallucinations were auditory hallucinations (35.9%), followed by visual hallucinations (28.1%).

**Table 1:** Characteristics of 64 patients with VLOSLP

	All	Not tested for DLB biomarkers (*n* = 30)	Tested for DLB biomarkers (*n* = 34)	Effect size	*p*^†^
Gender, female (%)	48 (75%)	20 (66.7%)	28 (83.4%)	0.181	0.148
Age, years	78.5 (6.4)	77.7 (6.2)	79.1 (6.5)	− 0.117	0.349
Onset age, years	75.8 (7.0)	75.3 (6.8)	76.2 (7.2)	− 0.090	0.471
Education duration, years	12.3 (2.2)	12.4 (2.5)	12.3 (1.9)	− 0.031	0.803
Cognitive battery					
MMSE	26.4 (1.8)	26.0 (1.7)	26.7 (1.8)	− 0.186	0.137
WMS-R LM I	10.7 (5.7)	9.4 (4.9)	11.8 (6.1)	− 0.187	0.136
WMS-R LM II	5.0 (4.5)	4.1 (4.2)	5.8 (4.7)	− 0.185	0.139
DSST, ss^a^	9.7 (3.2)	10.4 (3.0)	9.0 (3.2)	− 0.221	0.093
BDT, ss^b^	9.2 (3.1)	9.3 (2.9)	9.1 (3.3)	− 0.013	0.923
Digit span, ss^b^	10.7 (3.9)	10.9 (3.5)	10.5 (3.3)	− 0.048	0.718
Information, ss^b^	10.2 (2.4)	10.2 (2.3)	10.2 (2.4)	− 0.020	0.878
NPI-plus^c^					
Delusions	3.9 (4.0)	2.3 (2.9)	5.5 (4.3)	− 0.395^d^	0.002*
Hallucinations	2.3 (3.5)	0.8 (1.1)	3.7 (4.3)	− 0.375^d^	0.003*
Agitation/aggression	1.1 (2.7)	1.7 (3.1)	0.6 (2.3)	− 0.282	0.027*
Dysphoria/depression	0.7 (1.5)	0.5 (1.0)	0.9 (1.8)	− 0.043	0.733
Anxiety	1.2 (2.6)	0.7 (2.3)	1.7 (2.9)	− 0.246	0.053
Euphoria	0.1 (0.6)	0.2 (0.8)	0.0 (0.2)	− 0.087	0.495
Apathy	3.1 (3.7)	3.6 (3.5)	2.6 (3.9)	− 0.209	0.101
Disinhibition	1.0 (2.6)	1.2 (2.8)	0.8 (2.5)	− 0.130	0.308
Irritability	1.3 (2.9)	1.8 (3.4)	0.8 (2.3)	− 0.098	0.442
Aberrant motor behavior	0.4 (1.8)	0.2 (1.1)	0.5 (2.2)	− 0.067	0.596
Nighttime behavior	2.0 (2.9)	1.9 (3.0)	2.2 (2.8)	− 0.092	0.467
Appetite	1.7 (3.2)	2.3 (3.7)	1.2 (2.6)	− 0.148	0.244
Cognitive fluctuation	1.0 (2.0)	0.5 (1.3)	1.6 (2.4)	− 0.275	0.030*
Contents of delusions					
At least one delusion	49 (76.6%)	18 (60.0%)	31 (91.2%)	0.367^d^	0.003*
Delusion of persecution	26 (40.6%)	8 (26.7%)	18 (52.9%)	0.267	0.033*
Delusion of theft	25 (39.1%)	10 (33.3%)	15 (44.1%)	0.110	0.378
Delusional jealousy	6 (9.4%)	2 (6.7%)	4 (11.8%)	0.087	0.485
Phantom-border delusion	20 (31.3%)	5 (16.7%)	15 (44.1%)	0.296	0.018*
Misidentification of person	1 (1.6%)	0 (0.0%)	1 (2.9%)	0.118	0.344
Misidentification of place	2 (3.1%)	0 (0.0%)	2 (5.9%)	0.169	0.177
Delusion of abandonment	4 (6.3%)	3(10.0%)	1 (2.9%)	0.146	0.244
Misidentification of TV	3 (4.7%)	0 (0.0%)	3 (8.8%)	0.208	0.096
Other delusions	8 (12.5%)	2 (6.7%)	6 (17.6%)	0.166	0.185
Modalities of hallucinations					
At least one hallucination	35 (54.7%)	11 (36.7%)	24 (70.6%)	0.340^d^	0.007*
Auditory hallucinations	23 (35.9%)	8 (26.7%)	15 (44.1%)	0.181	0.147
Monolog	10 (15.6%)	3(10.0%)	7 (20.6%)	0.146	0.244
Visual hallucinations	18 (28.1%)	3(10.0%)	15 (44.1%)	0.379^d^	0.002*
Hallucinations of smell	3 (4.7%)	2 (6.7%)	1 (2.9%)	0.088	0.482
Tactile hallucinations	2 (3.1%)	1 (3.3%)	1 (2.9%)	0.011	0.928
Hallucinations of taste	0 (0.0%)	0 (0.0%)	0 (0.0%)	-	-
Other hallucinations	4 (6.3%)	1 (3.3%)	3 (8.8%)	0.113	0.365
Medications					
Antipsychotics	12 (18.8%)	5 (16.7%)	7 (20.6%)	0.050	0.688
Benzodiazepine	22 (34.4%)	10 (33.3%)	12 (35.3%)	0.021	0.869
Anticholinergic drugs	2 (3.1%)	1 (3.3%)	1 (2.9%)	0.011	0.928

Data represent mean (SD) or number (%)

*Abbreviations*: *VLOSLP* very late-onset schizophrenia-like psychosis, *DLB* dementia with Lewy bodies, *MMSE* Mini-Mental State Examination, *WMS-R* Wechsler Memory Scale-Revised, *LM* logical memory, *DSST* Digit Symbol Substitution Test, *BDT* block design test, *ss* standardized score, *NPI* Neuropsychiatric Inventory

^†^Results of the Mann–Whitney *U* test for continuous and ordinal variables and chi-square test for nominal variables

*p < 0.05

^a^Lack of data on two patients in VLOSLP without DLB biomarkers and on four patients in VLOSLP with DLB biomarkers

^b^Lack of data on two patients in VLOSLP without DLB biomarkers and on five patients in VLOSLP with DLB biomarkers

^c^Lack of data on two patients in VLOSLP with DLB biomarkers

^d^Medium-to-large effect size. We calculated *r* for Mann-Whitney *U* test and Cohen’s *W* for chi-square test as effect size

Thirty-four patients underwent testing for at least one of the indicative biomarkers of DLB, of which 17 underwent DAT SPECT and 22 underwent MIBG myocardial scintigraphy. Table 1 shows the differences between patients who underwent DLB biomarkers and those who did not. The scores of delusions (*p* = 0.002), hallucinations (*p* = 0.003), and cognitive fluctuation (*p* = 0.003) in the NPI-plus were significantly higher, and the scores for agitation/aggression (*p* = 0.027) were significantly lower in those who underwent indicative biomarkers than in those who did not. Delusions of persecution (53.9% vs. 26.7%, *p* = 0.033), phantom-border delusions (44.1% vs. 16.7%, *p* = 0.018), and visual hallucinations (44.1% vs. 10.0%, *p* = 0.002) were more prevalent in patients who underwent testing for indicative biomarkers than in those who did not. There were no differences in age, gender, or cognitive battery between the groups.

### VLOSLP and results of indicative biomarkers of DLB

Among 34 patients who underwent testing for indicative biomarkers of DLB, 17 patients underwent DAT SPECT and eight showed positive results, while 22 underwent MIBG myocardial scintigraphy and six showed positive results. Five patients underwent testing for both indicative biomarkers; three were positive in both, one was negative in both, and one was positive in MIBG myocardial scintigraphy but negative in DAT SPECT. Therefore, 11 patients (32.4%) showed positive results for the indicative biomarkers (VLOSLP+LB).

**Fig. 2:**
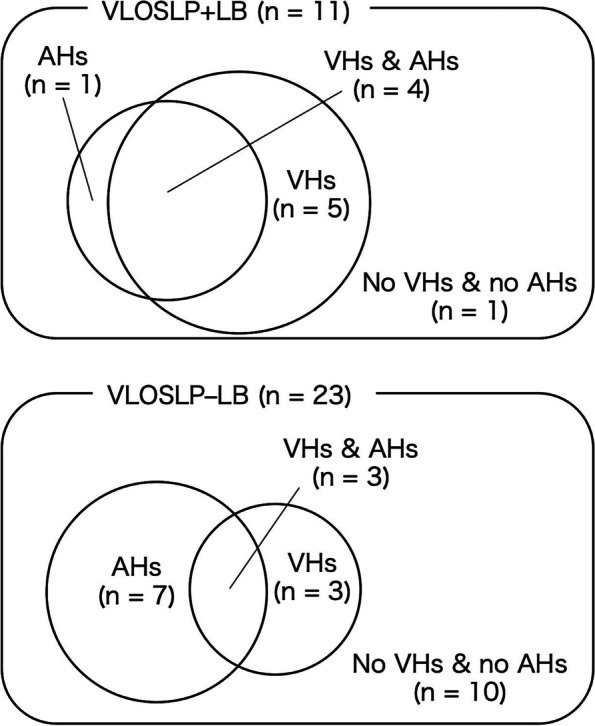
Overlap of visual and auditory hallucinations. VLOSLP, very late-onset schizophrenia-like psychosis; DLB, dementia with Lewy bodies; VLOSLP+LB, VLOSLP with positive results in indicative biomarkers of DLB; VLOSLP–LB, VLOSLP with negative results in indicative biomarkers of DLB; VHs, visual hallucinations; AHs, auditory hallucinations. One patient in VLOSLP+LB and 10 patients in VLOSLP–LB complained of neither visual nor auditory hallucinations, but they all had various delusions, and one of them also complained of hallucinations of “feeling energy

The standardized score of DSST (mean [SD] score = 6.9 [3.1] vs. 10.0 [2.7], *p* = 0.005) and the score of delusions in the NPI-plus (mean [SD] score = 2.4 [2.3] vs. 6.9 [4.3], *p* = 0.005) were significantly lower in the VLOSLP+LB group than in the VLOSLP–LB group (Table 2). There were no differences in gender, age, and other cognitive battery between the two groups. Visual hallucinations were significantly more prevalent in the VLOSLP+LB group than in the VLOSLP–LB group (81.8% vs. 26.1%, *p* = 0.002), although the prevalence of delusions and other hallucinations between the groups was not different. Auditory hallucinations were prevalent in both groups (43.5% in VLOSLP–LB, and 45.5% in VLOSLP+LB). Among patients with auditory hallucinations, AH–VH tended to be more prevalent in VLOSLP–LB (7 out of 10) than in VLOSLP+LB patients (1 out of 5) (Fig. 2).

**Table 2:** Comparison between VLOSLP patients with positive results and negative results in tests for indicative biomarkers of DLB

	VLOSLP+LB (*n* = 11)	VLOSLP–LB (*n* = 23)	Effect size	*p*^†^
Gender, female (%)	8 (72.7%)	20 (87.0%)	0.175	0.309
Age, years	76.8 (7.4)	80.2 (5.9)	− 0.228	0.188
Onset age, years	74.5 (7.8)	77.1 (7.0)	− 0.158	0.363
Education duration, years	12.3 (1.7)	12.3 (2.0)	− 0.030	0.885
Cognitive battery				
MMSE	26.2 (1.9)	26.9 (1.7)	− 0.182	0.308
WMS-R LM I	10.5 (7.4)	12.4 (5.5)	− 0.237	0.178
WMS-R LM II	5.5 (4.2)	5.9 (5.1)	− 0.016	0.925
DSST, ss^a^	6.9 (3.1)	10.0 (2.7)	− 0.510^d^	0.005*
BDT, ss^b^	7.4 (3.2)	9.9 (3.1)	− 0.358^d^	0.055
Digit span, ss^b^	10.0 (3.7)	10.8 (3.1)	− 0.097	0.627
Information, ss^b^	10.6 (2.1)	10.1 (2.6)	− 0.174	0.365
NPI-plus^c^				
Delusions	2.4 (2.3)	6.9 (4.3)	− 0.482^d^	0.005*
Hallucinations	2.7 (2.3)	4.1 (5.0)	− 0.051	0.795
Agitation/aggression	0.0 (0.0)	0.9 (2.7)	− 0.213	0.562
Dysphoria/depression	0.3 (0.7)	1.1 (2.1)	− 0.159	0.483
Anxiety	1.3 (2.7)	1.9 (3.0)	− 0.104	0.617
Euphoria	0.0 (0.0)	0.0 (0.2)	− 0.119	0.857
Apathy	0.8 (1.7)	3.5 (4.3)	− 0.308^d^	0.129
Disinhibition	0.1 (0.3)	1.2 (3.0)	− 0.125	0.675
Irritability	0.4 (1.0)	1.0 (2.7)	− 0.050	0.857
Aberrant motor behavior	0.0 (0.0)	0.7 (2.7)	− 0.171	0.704
Nighttime behavior	0.6 (1.3)	2.9 (3.0)	− 0.352^d^	0.064
Appetite	0.6 (1.9)	1.5 (2.9)	− 0.153	0.562
Cognitive fluctuation	1.4 (2.1)	1.6 (2.5)	− 0.008	0.984
Contents of delusions				
At least one delusion	9 (81.8%)	22 (95.7%)	0.228	0.183
Delusion of persecution	4 (36.4%)	14 (60.9%)	0.230	0.180
Delusion of theft	4 (36.4%)	11 (47.8%)	0.108	0.529
Delusional jealousy	1 (9.1%)	3 (13.0%)	0.057	0.738
Phantom-border delusion	6 (54.5%)	9 (39.1%)	0.145	0.397
Misidentification of person	0 (0.0%)	1 (4.3%)	0.120	0.483
Misidentification of place	0 (0.0%)	2 (8.7%)	0.173	0.313
Delusion of abandonment	0 (0.0%)	1 (4.3%)	0.120	0.483
Misidentification of TV	0 (0.0%)	3 (13.0%)	0.215	0.210
Other delusions	1 (9.1%)	5 (21.7%)	0.155	0.365
Modalities of hallucinations				
at least one hallucination	10 (90.9%)	14 (60.9%)	0.308^d^	0.072
Auditory hallucinations	5 (45.5%)	10 (43.5%)	0.019	0.914
Monolog	3 (27.3%)	4 (17.4%)	0.114	0.505
Visual hallucinations	9 (81.8%)	6 (26.1%)	0.525^d^	0.002*
Hallucinations of smell	0 (0.0%)	1 (4.3%)	0.120	0.483
Tactile hallucinations	0 (0.0%)	1 (4.3%)	0.120	0.483
Hallucinations of taste	0 (0.0%)	0 (0.0%)	-	-
Other hallucinations	1 (9.1%)	2 (8.7%)	0.005	0.970
Medications				
Antipsychotics	2 (18.2%)	5 (21.7%)	0.041	0.810
Benzodiazepine	3 (27.3%)	9 (39.1%)	0.116	0.498
Anticholinergic drugs	1 (9.1%)	0 (0.0%)	0.252	0.142

Data represent mean (SD) or number (%)

*Abbreviations*: *VLOSLP* very late-onset schizophrenia-like psychosis, *DLB* dementia with Lewy bodies, *VLOSLP+LB* VLOSLP with positive results as indicative biomarkers of DLB, *VLOSLP–LB* VLOSLP with negative results in indicative biomarkers of DLB, *MMSE* Mini-Mental State Examination, *WMS-R* Wechsler Memory Scale-Revised, *LM* logical memory, *DSST* Digit Symbol Substitution Test, *BDT* block design test, *ss* standardized score, *NPI* Neuropsychiatric Inventory

^†^Results of Mann-Whitney *U* test for continuous and ordinal variables and chi-square test for nominal variables

*p < 0.05

^a^Lack of data on a patient in VLOSLP+LB and on three patients in VLOSLP–LB

^b^Lack of data on two patients in VLOSLP+LB and on three patients in VLOSLP–LB

^c^Lack of data on a patient in VLOSLP+LB and on a patient in VLOSLP–LB

^d^Medium-to-large effect size. We calculated *r* for Mann-Whitney *U* test and Cohen’s *W* for chi-square test as effect size

### Gray matter volume and cerebral blood flow between VLOSLP+LB and VLOSLP–LB

Ten patients in the VLOSLP+LB group and 14 in VLOSLP–LB group underwent 3D T1 MRI. Gray matter volume was not significantly different in any region between the VLOSLP+LB and VLOSLP–LB groups.

**Fig. 3:**
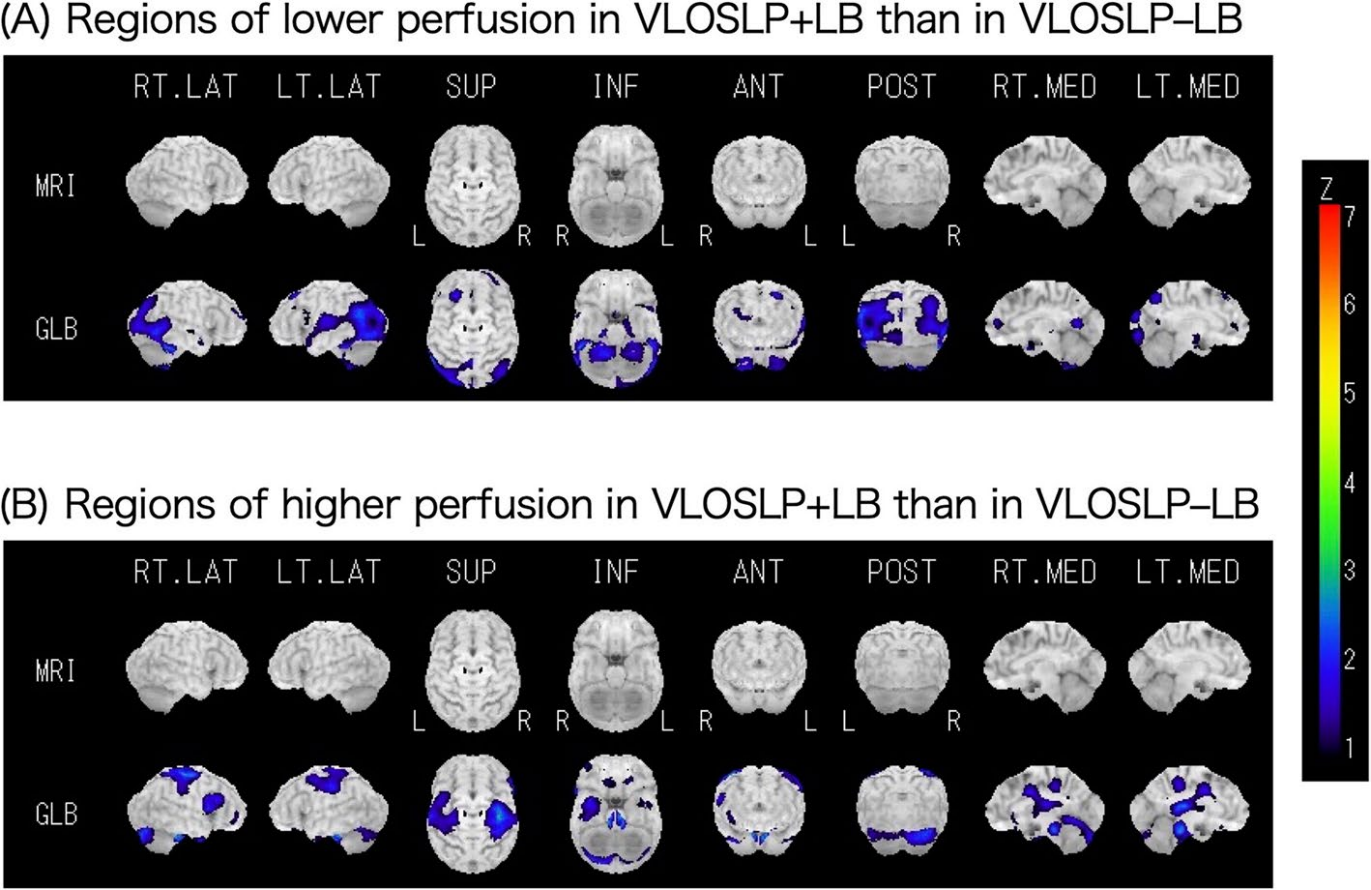
Comparison of cerebral perfusion between VLOSLP+LB and VLOSLP–LB. Color coding represents the statistical significance (Z-score) of the lower (**A**) or higher (**B**) perfusion in VLOSLP+LB than in VLOSLP–LB. VLOSLP, very late-onset schizophrenia-like psychosis; DLB, dementia with Lewy bodies; VLOSLP+LB, VLOSLP with positive results in indicative biomarkers of DLB; VLOSLP–LB, VLOSLP with negative results in indicative biomarkers of DLB

Eleven patients in the VLOSLP+LB group and 16 in the VLOSLP–LB group underwent ^123^I IMP-SPECT. Cerebral blood flow was lower in VLOSLP+LB patients as compared to VLOSLP–LB patients in the posterior regions, including the occipital lobe (Fig. 3). Cerebral blood flow was higher in the bilateral precentral gyrus, postcentral gyrus, cingulate, thalamus, brainstem, and right temporal lobe in the VLOSLP+LB group than in the VLOSLP–LB group.

## Discussion

In the present study, we confirmed that some patients with VLOSLP showed positive results for the indicative biomarkers of DLB. Lower scores of DSST, lower scores of delusions in the NPI, and a high prevalence of visual hallucinations are the features of VLOSLP+LB patients as compared to VLOSLP–LB patients, although the clinical characteristics were similar between the two groups. There was no significant difference in gray matter volume between the two groups, and cerebral blood flow in the extent area including the occipital lobe was lower in VLOSLP+LB patients than in VLOSLP–LB patients, which is consistent with the findings of supportive biomarkers of DLB.

The prevalence of positive results for indicative biomarkers was 11 of 34 (32.4%) patients who underwent testing for these biomarkers. A previous neuropathological study reported that four (36.4%) of 11 patients with late-onset schizophrenia and delusional disorder with onset at ≥ 65 years had Lewy body pathology [6], which is consistent with the present results. Among the patients with VLOSLP, cognitive fluctuation was more severe, and phantom-border delusions and visual hallucinations were more prevalent in those who underwent testing for indicative biomarkers of DLB than in those who did not. Since these symptoms are characteristic of DLB, our results mean that we tended to use the indicative biomarkers in patients with VLOSLP who presented with symptoms suggestive of DLB. Therefore, the positive rate in patients with VLOSLP may be lower than the results of this study. However, even if the results of all the patients who were not tested for the indicative biomarkers were negative, the positive rate in the present study was at least 11 of 64 (17.2%), suggesting that there are a certain number of patients who are suspected of prodromal DLB among those with VLOSLP.

The DSST score of VLOSLP+LB patients was worse than those of VLOSLP–LB patients. The results suggest that psychomotor slowing detected with DSST is suggestive of prodromal DLB in VLOSLP. A previous study reported that a low DSST score predicts clinical cognitive disorders in healthy older adults [25]. Psychomotor slowing may be a predictive feature of cognitive impairment that is common in healthy older people and patients with VLOSLP. From another point of view, it is often difficult to differentiate psychomotor slowing from bradykinesia [10]. The low DSST score in VLOSLP+LB might be caused by bradykinesia due to subtle Parkinsonism that could not be clearly identified.

Visual hallucinations were more prevalent in VLOSLP+LB patients than in those with VLOSLP–LB, which was reasonable considering that visual hallucinations are one of the core clinical features of DLB. However, since 26.1% of VLOSLP–LB also had visual hallucinations, the presence of visual hallucinations alone is not sufficient to indicate prodromal DLB in VLOSLP. In both VLOSLP+LB and VLOSLP–LB, auditory hallucinations were present in approximately 45% of the patients. A previous study on DLB reported that auditory hallucinations were found in approximately 35% of patients with DLB and that most of those with auditory hallucinations were accompanied by visual hallucinations [21]. In the present study, among five patients with auditory hallucinations in the VLOSLP+LB group, four patients had accompanying visual hallucinations; however, only three patients had visual hallucinations among 10 patients with auditory hallucinations in the VLOSLP–LB group, suggesting that auditory hallucinations in isolation are unlikely to predict further evolution to DLB in patients with VLOSLP.

While comparing the MRI results between the VLOSLP+LB and VLOSLP–LB groups, there were no areas that showed significant volume differences. In contrast, cerebral blood flow was lower in the posterior regions, including the occipital lobe and was higher in the bilateral precentral gyrus, postcentral gyrus, cingulate, thalamus, brainstem, and right temporal lobe in VLOSLP+LB patients as compared to VLOSLP–LB patients. These results agree with “generalized low perfusion with reduced occipital activity,” which are supportive biomarkers of DLB [26]. In addition, the areas with relatively high perfusion in VLOSLP+LB patients correspond to those with relatively increased glucose metabolism reported as Parkinson’s disease-related pattern in Parkinson’s disease [27] and those with a cingulate island sign in DLB [28]. The present pattern of brain perfusion seen in VLOSLP+LB patients compared to those with VLOSLP–LB is consistent with the characteristics of those in LBD reported in previous studies.

In both VLOSLP+LB and VLOSLP–LB patients, females were overwhelmingly represented, which is consistent with the findings of VLOSLP [4, 29]. In a study of pathologically defined dementia with Lewy bodies, fewer females had visual hallucinations compared with males [30]. This seems to contradict the present result of there being more females in the VLOSLP+LB group. On the other hand, a retrospective observational study of DLB reported that there were no gender differences in those with visual hallucinations or delusions as their first symptom but that there were more females in those with auditory hallucinations as their first symptom [31]. A recent study reported that visual hallucinations appeared earlier and concurrently with the other core clinical features in female patients with probable DLB; however, visual hallucinations developed after the other core clinical features in male patients [32]. The results of these previous studies and the present study suggest that there are more females than males among patients with VLOSLP as prodromal DLB although the incidence of visual hallucinations in patients with DLB over the entire period may be higher in men than in women.

Based on the results discussed above, it is suggested that VLOSLP+LB patients show features typical of LBD compared to those with VLOSLP–LB. Characteristics such as psychomotor slowing and visual hallucinations may suggest prodromal DLB in VLOSLP patients. However, there were no statistically significant differences between the two groups in the measures of cognition and neuropsychiatric symptoms other than DSST, delusions score of NPI, and prevalence of visual hallucinations. The common characteristics of highly prevalent auditory/visual hallucinations and persecution delusions, as well as a high rate of females, in both groups were typical features of VLOSLP that have been consistently reported [4, 29, 33]. There may be many cases of difficulty in distinguishing between VLOSLP+LB and VLOSLP–LB on clinical symptoms alone. Although the efficacy of antipsychotics on VLOSLP has been reported [11], hypersensitivity to antipsychotics is often seen in patients with DLB [26]. Therefore, differentiating between VLOSLP+LB and VLOSLP–LB may be important in treatment selection. Biomarkers of DLB would be helpful to those with VLOSLP.

The present results revealed that a certain number of patients with VLOSLP are suspected to have LBD even when they do not present with dementia or Parkinsonism. The strength of the current study was the use of indicative biomarkers of DLB when patients presented with VLOSLP to evaluate the presence and characteristics of VLOSLP+LB. Since previous studies retrospectively assessed the presence of LBD in VLOSLP based on postmortem neuropathology, it was not possible to examine the detailed clinical manifestations and the presence or absence of Lewy pathology at the time of VLOSLP diagnosis.

### Limitations

This study has some limitations. It should be noted that no biomarker directly assessed the presence of Lewy pathology. Although the utility of both imaging in distinguishing DLB from AD is well-established with high sensitivity and specificity [34, 35], the utility for the prodromal stage of DLB is not very high [16, 17]. A previous neuropathological study reported that some patients with late-onset schizophrenia and delusional disorders, which overlap with VLOSLP, had corticobasal degeneration [6], which may have caused the reduced uptake on DAT SPECT [36, 37]. In addition, neurodegenerations other than LBD, such as argyrophilic grain disease and primary age-related tauopathy, are also reportedly associated with VLOSLP [5, 6]. Some case reports have shown AD pathology in patients with VLOSLP [38, 39]. We should consider the rate of false positives and false negatives of indicative biomarkers and the influence of neurodegeneration other than LBD in interpreting the results.

There are other limitations in addition to the selection bias in conducting neuroimaging tests, the characteristics of biomarkers, and the influence of other neurodegenerations as described above. First, the sample size was too small to perform multivariate analysis. Second, this was a retrospective, cross-sectional, single-center study. Third, we did not control confounders of psychosis such as medications and sensory impairment. Finally, we did not conduct statistical correction for multiple comparisons. We considered this study to be at the stage of exploring the characteristics of patients suggestive of prodromal DLB in VLOSLP. Therefore, we focused on avoiding the risk of β-error rather than α-error in this small-sample-size study. For this reason, we decided that it would be preferable to present the results without correction for multiple comparisons. In fact, many of the items that were judged to be significantly different and some of the items that were judged to be not significantly different showed medium-to-large effect sizes. Further longitudinal prospective studies with larger sample sizes are required.

## Conclusions

There are a certain number of patients with positive results for indicative biomarkers of DLB among those with VLOSLP. These patients may present with psychosis as a prodromal DLB. Psychomotor slowing, visual hallucinations, and reduced perfusion in occipital lobe may be suggestive of prodromal DLB in VLOSLP, although the clinical manifestations were similar in many respects between VLOSLP+LB and VLOSLP–LB patients.

## Data Availability

The datasets used and/or analyzed during the current study are available from the corresponding author on reasonable request.

## Abbreviations

VLOSLP: Very-late-onset schizophrenia-like psychosis
LBD: Lewy body disease
DLB: Dementia with Lewy bodies
AD: Alzheimer’s disease
MIBG: ^123^I-meta-iodobenzylguanidine
MCI: Mild cognitive impairment
RBD: REM sleep behavior disorder
NPI: Neuropsychiatric Inventory
BPRS: Brief Psychiatric Rating Scale
MMSE: Mini-Mental State Examination
CDR: Clinical Dementia Rating
MRI: Magnetic resonance imaging
DSST: Digit Symbol Substitution Test
WAIS-III: Wechsler Adult Intelligence Scale-III
AH+VH: Auditory hallucinations were accompanied with visual hallucinations
AH–VH: Auditory hallucinations were accompanied without visual hallucinations
DAT: Dopamine transporter
SPECT: Single-photon emission computed tomography
VLOSLP+LB: VLOSLP with positive results as indicative biomarkers of DLB
VLOSLP–LB: VLOSLP with negative results as indicative biomarkers of DLB
SPM: Statistical Parametric Mapping
DARTEL: Diffeomorphic anatomic registration through exponentiated lie algebra
^123^I-IMP: N-isopropyl-p-[^123^I] iodoamphetamine

## References

[1] UN Decade of Healthy Ageing. [cited 2021 Oct 24]. Available from: https://www.who.int/initiatives/decade-of-healthy-ageing

[2] Kay DW, Roth M (1961). Environmental and hereditary factors in the schizophrenias of age (“late paraphrenia”) and their bearing on the general problem of causation in schizophrenia. J Ment Sci.

[3] Pearlson GD, Kreger L, Rabins PV, Chase GA, Cohen B, Wirth JB (1989). A chart review study of late-onset and early-onset schizophrenia. Am J Psychiatry.

[4] Howard R, Rabins PV, Seeman MV, Jeste DV (2000). Late-onset schizophrenia and very-late-onset schizophrenia-like psychosis: an international consensus. The International Late-Onset Schizophrenia Group. Am J Psychiatry.

[5] Casanova MF, Stevens JR, Brown R, Royston C, Bruton C (2002). Disentangling the pathology of schizophrenia and paraphrenia. Acta Neuropathol.

[6] Nagao S, Yokota O, Ikeda C, Takeda N, Ishizu H, Kuroda S (2014). Argyrophilic grain disease as a neurodegenerative substrate in late-onset schizophrenia and delusional disorders. Eur Arch Psychiatry Clin Neurosci.

[7] Brodaty H, Sachdev P, Koschera A, Monk D, Cullen B (2003). Long-term outcome of late-onset schizophrenia: 5-year follow-up study. Br J Psychiatry.

[8] Kørner A, Lopez AG, Lauritzen L, Andersen PK, Kessing LV (2009). Late and very-late first-contact schizophrenia and the risk of dementia--a nationwide register based study. Int J Geriatr Psychiatry.

[9] Stafford J, Dykxhoorn J, Sommerlad A, Dalman C, Kirkbride JB, Howard R. Association between risk of dementia and very late-onset schizophrenia-like psychosis: a Swedish population-based cohort study. Psychol Med. 2021:1–9. https://www.cambridge.org/core/journals/psychological-medicine/article/association-between-risk-of-dementia-and-very-lateonset-schizophrenialike-psychosis-a-swedish-populationbased-cohort-study/2F6BC9F9D4D544D1BD081B061E04F1C2. 10.1017/S0033291721002099 PMC9975996 34030750

[10] McKeith IG, Ferman TJ, Thomas AJ, Blanc F, Boeve BF, Fujishiro H (2020). Research criteria for the diagnosis of prodromal dementia with Lewy bodies. Neurology..

[11] Howard R, Cort E, Bradley R, Harper E, Kelly L, Bentham P (2018). Antipsychotic treatment of very late-onset schizophrenia-like psychosis (ATLAS): a randomised, controlled, double-blind trial. Lancet Psychiatry.

[12] Thomas AJ, Donaghy P, Roberts G, Colloby SJ, Barnett NA, Petrides G (2019). Diagnostic accuracy of dopaminergic imaging in prodromal dementia with Lewy bodies. Psychol Med.

[13] Fujishiro H, Okuda M, Iwamoto K, Miyata S, Torii Y, Iritani S (2018). Early diagnosis of Lewy body disease in patients with late-onset psychiatric disorders using clinical history of rapid eye movement sleep behavior disorder and [123 I]-metaiodobenzylguanidine cardiac scintigraphy. Psychiatry Clin Neurosci.

[14] Kobayashi K, Nakano H, Akiyama N, Maeda T, Yamamori S (2015). Pure psychiatric presentation of the Lewy body disease is depression--an analysis of 60 cases verified with myocardial meta-iodobenzylguanidine study. Int J Geriatr Psychiatry.

[15] Takahashi S, Mizukami K, Arai T, Ogawa R, Kikuchi N, Hattori S (2016). Ventilatory response to hypercapnia predicts dementia with Lewy bodies in late-onset major depressive disorder. J Alzheimers Dis.

[16] Roberts G, Donaghy PC, Lloyd J, Durcan R, Petrides G, Colloby SJ (2021). Accuracy of dopaminergic imaging as a biomarker for mild cognitive impairment with Lewy bodies. Br J Psychiatry.

[17] Roberts G, Durcan R, Donaghy PC, Lawley S, Ciafone J, Hamilton CA (2021). Accuracy of cardiac innervation scintigraphy for mild cognitive impairment with Lewy bodies. Neurology..

[18] Suehiro T, Satake Y, Hashimoto M, Ikeda M (2021). Case report: De Clerambault’s syndrome in dementia with Lewy bodies. Front Psych.

[19] Mori E, Ikeda M, Kosaka K (2012). Donepezil-DLB Study Investigators. Donepezil for dementia with Lewy bodies: a randomized, placebo-controlled trial. Ann Neurol.

[20] Cummings JL (1997). The Neuropsychiatric Inventory: assessing psychopathology in dementia patients. Neurology..

[21] Tsunoda N, Hashimoto M, Ishikawa T, Fukuhara R, Yuki S, Tanaka H, et al. Clinical features of auditory hallucinations in patients with dementia with Lewy bodies: a soundtrack of visual hallucinations. J Clin Psychiatry. 2018;79(3):17m11623. 10.4088/JCP.17m11623 29742332

[22] Matsuda H, Murata M, Mukai Y, Sako K, Ono H, Toyama H (2018). Japanese multicenter database of healthy controls for [123I]FP-CIT SPECT. Eur J Nucl Med Mol Imaging.

[23] Nakajima K, Matsumoto N, Kasai T, Matsuo S, Kiso K, Okuda K (2016). Normal values and standardization of parameters in nuclear cardiology: Japanese Society of Nuclear Medicine working group database. Ann Nucl Med.

[24] Minoshima S, Frey KA, Koeppe RA, Foster NL, Kuhl DE (1995). A diagnostic approach in Alzheimer’s disease using three-dimensional stereotactic surface projections of fluorine-18-FDG PET. J Nucl Med.

[25] Rosano C, Perera S, Inzitari M, Newman AB, Longstreth WT, Studenski S (2016). Digit Symbol Substitution test and future clinical and subclinical disorders of cognition, mobility and mood in older adults. Age Ageing.

[26] McKeith IG, Boeve BF, Dickson DW, Halliday G, Taylor JP, Weintraub D (2017). Diagnosis and management of dementia with Lewy bodies: fourth consensus report of the DLB Consortium. Neurology..

[27] Ma Y, Tang C, Spetsieris PG, Dhawan V, Eidelberg D (2007). Abnormal metabolic network activity in Parkinson’s disease: test-retest reproducibility. J Cereb Blood Flow Metab.

[28] Lim SM, Katsifis A, Villemagne VL, Best R, Jones G, Saling M (2009). The 18F-FDG PET cingulate island sign and comparison to 123I-beta-CIT SPECT for diagnosis of dementia with Lewy bodies. J Nucl Med.

[29] Mason O, Stott J, Sweeting R (2013). Dimensions of positive symptoms in late versus early onset psychosis. Int Psychogeriatr.

[30] Bayram E, Coughlin DG, Banks SJ, Litvan I (2021). Sex differences for phenotype in pathologically defined dementia with Lewy bodies. J Neurol Neurosurg Psychiatry.

[31] Utsumi K, Fukatsu R, Yamada R, Takamaru Y, Hara Y, Yasumura S (2020). Characteristics of initial symptoms and symptoms at diagnosis in probable dementia with Lewy body disease: incidence of symptoms and gender differences. Psychogeriatrics..

[32] Choudhury P, Graff-Radford J, Aakre JA, Wurtz L, Knopman DS, Graff-Radford NR (2022). The temporal onset of the core features in dementia with Lewy bodies. Alzheimers Dement.

[33] Girard C, Simard M (2008). Clinical characterization of late- and very late-onset first psychotic episode in psychiatric inpatients. Am J Geriatr Psychiatry.

[34] Yoshita M, Arai H, Arai H, Arai T, Asada T, Fujishiro H (2015). Diagnostic accuracy of 123I-meta-iodobenzylguanidine myocardial scintigraphy in dementia with Lewy bodies: a multicenter study. PLoS One.

[35] McKeith I, O’Brien J, Walker Z, Tatsch K, Booij J, Darcourt J (2007). Sensitivity and specificity of dopamine transporter imaging with 123I-FP-CIT SPECT in dementia with Lewy bodies: a phase III, multicentre study. Lancet Neurol.

[36] Plotkin M, Amthauer H, Klaffke S, Kühn A, Lüdemann L, Arnold G (2005). Combined 123I-FP-CIT and 123I-IBZM SPECT for the diagnosis of parkinsonian syndromes: study on 72 patients. J Neural Transm (Vienna).

[37] Brooks DJ (2016). Molecular imaging of dopamine transporters. Ageing Res Rev.

[38] Fujishiro H, Iritani S, Hattori M, Sekiguchi H, Matsunaga S, Habuchi C (2015). Autopsy-confirmed hippocampal-sparing Alzheimer’s disease with delusional jealousy as initial manifestation. Psychogeriatrics..

[39] Satake Y, Kanemoto H, Yoshiyama K, Nakahama R, Matsunaga K, Shimosegawa E (2021). Case Report: Usefulness of biomarkers for Alzheimer’s disease in two cases with very-late-onset schizophrenia-like psychosis. Front Psych.

